# A simple and effective method for ultrastructural analysis of mitosis in *Drosophila* S2 cells

**DOI:** 10.1016/j.mex.2016.10.003

**Published:** 2016-10-18

**Authors:** Anton Strunov, Lidiya V. Boldyreva, Gera A. Pavlova, Alexey V. Pindyurin, Maurizio Gatti, Elena Kiseleva

**Affiliations:** a Institute of Molecular and Cellular Biology, Siberian Branch of RAS, Novosibirsk, 630090, Russia; b Institute of Cytology and Genetics, Siberian Branch of RAS, Novosibirsk, 630090, Russia; c Kazan Federal University, Kazan, 420008, Russia; d Novosibirsk State University, Novosibirsk, 630090, Russia; e IBPM CNR and Department of Biology and Biotechnology, Sapienza University of Rome, Rome, 00185, Italy

**Keywords:** A simple and effective method for ultrastructural analysis of mitosis in Drosophila S2 cells, *Drosophila* S2 cells, Unsynchronized cell cultures, Mitosis, Transmission electron microscopy, Ultrastructural analysis

## Abstract

The *Drosophila* S2 tissue culture cells are a widely used system for studies on mitosis. S2 cells are particularly sensitive to gene silencing by RNA interference (RNAi), allowing targeted inactivation of mitotic genes. S2 cells are also well suited for high-resolution light microscopy analysis of mitosis in fixed cells, and can be easily immunostained to detect mitotic components. In addition, S2 cells are amenable to transformation with plasmid encoding fluorescently tagged mitotic proteins, allowing *in vivo* analysis of their behavior throughout cell division. However, S2 cells have not been widely used for transmission electron microscopy (TEM) analysis, which provides ultrastructural details on the morphology of the mitotic apparatus that cannot be obtained with high-resolution confocal microscopy. Here, we describe a simple method for the ultrastructural analysis of mitosis in *Drosophila* S2 cells.

• Our method, which involves fixation and sectioning of a cell pellet, provides excellent preservation of mitotic structures and allows analysis of a higher number of mitotic divisions per sample, compared to correlative light-electron microscopy.
• Dividing cells are randomly oriented within the pellet and are sectioned along different planes, providing all-around information on the structure of the mitotic apparatus.

## Graphical abstract

**Figure:**
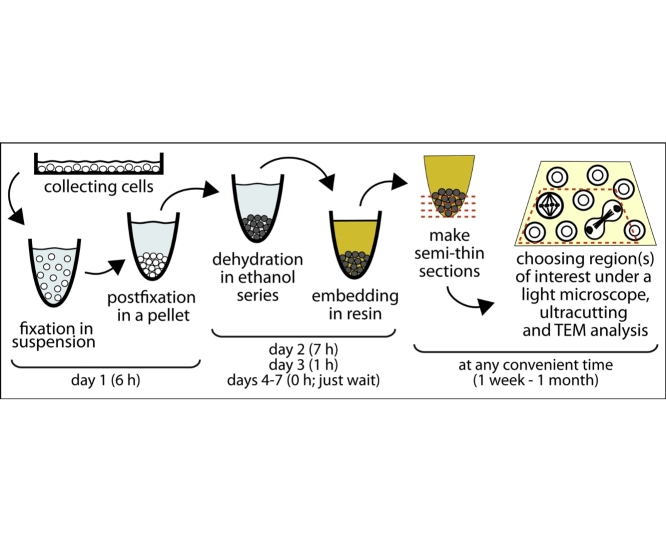

## Method details

### Preliminary notes

All procedures should be performed at room temperature (23 ± 2 °C) unless otherwise specified. Refrigerated solutions should be allowed to reach room temperature before use. If more than one specimen is processed, the number of cells and all the volumes of solutions should be adjusted accordingly. Each phase of the protocol is carried out without pauses, permitting a precise estimation of the experimental timing beforehand. Cell harvesting and fixation procedures take 5–6 h and are followed by an overnight post-fixation step. Drying the specimen and its embedding in a resin takes the next 2 days, followed by 2–3 additional days required for resin polymerization. The resin-embedded specimen can be then stored indefinitely at room temperature before sectioning.

### Cell culture handling

The S2 cells used here have been grown in the laboratory of one of the authors (MG) since 1997 and have been employed in several RNAi-based studies (e.g., [1], [2]). Since 1997, the line has been frozen 4 times. After each thawing, the cells have been propagated for 2–3 months and frozen again. The cells examined in this study are from aliquots frozen in 2004 (fourth freezing) in the laboratory of MG and cultured for 2 months at the IMCB in Novosibirsk. The karyotype of our S2 cells is slightly different from those of the S2-DRSC and S2R+ cells, although the three lines share several marker chromosomes [2], [3], [4]. It is therefore unlikely that our S2 line is a derivative of the S2R+ line, which has been first described in 1998 [5]. In addition, our S2 cells do not grow attached to the surface of the flasks as do the S2R+ cells. Thus, we believe that our S2 cell line is one of the many sub-lines derived from the original Schneider’s 2 line [6].

**Fig. 1:**
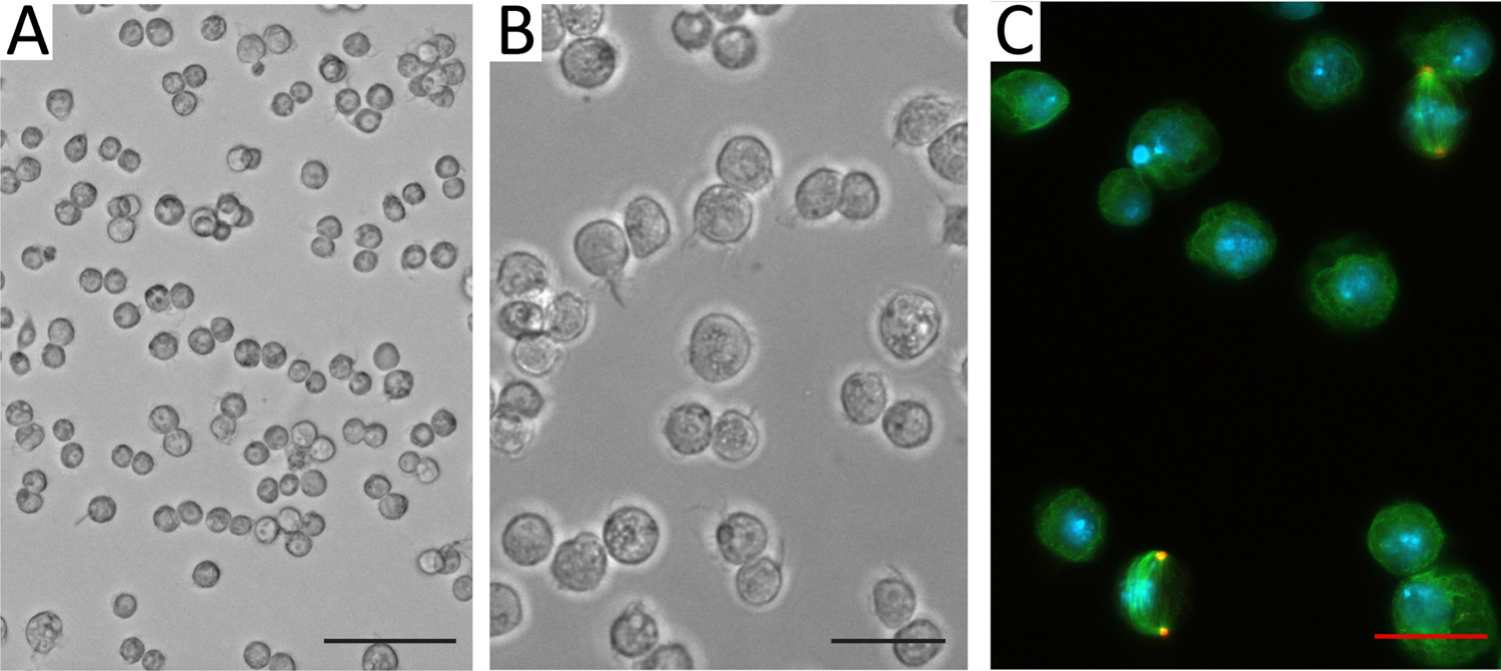
(A, B) Live *Drosophila* S2 cells in culture as seen under a phase-contrast light microscope (A and B, 20× and 40× magnification, respectively). (С) Fixed metaphase and interphase cells obtained according to [2]. Cells are stained with DAPI (DNA, blue), anti-α-tubulin antibodies (green) and antibodies against the centrosomal marker DSpd2 (red) [8] (40× magnification). Scale bars: (A), 50 μm; (B, C), 20 μm (For interpretation of the references to colour in this figure legend, the reader is referred to the web version of this article.).

Cells are maintained at 25 °C in Schneider’s Insect Medium (Sigma #S0146) supplemented with 10% Fetal Bovine Serum (Gibco #10270; heat-inactivated for 1 h at 65 °C). The cell density should be kept below 6–8 × 10^6^ cells/ml to avoid formation of cell aggregates (Fig. 1) [7]. To increase the proportion of dividing cells, it is advisable to split the culture 1:2 with fresh medium one day before the fixation procedure. The mitotic index (percentage of mitotic cells) in the specimens used to illustrate the protocol was 6.05 ± 0.77 (mean ± SEM; calculated by examining samples of 1000 cells from 4 independent experiments).

### Fixation of cell suspension

Cells should be harvested immediately before fixation and treated gently (both the suspension and the pellet) during all steps. The optimal cell number per specimen is 4–5 × 10^6^. The prefixation solution (2.5% glutaraldehyde in the culture medium) should be prepared immediately before addition to the cell suspension: 1 volume of 25% glutaraldehyde stock solution (Sigma #G5882) is added to 9 volumes of the culture medium (see “Cell culture handling”). It is crucial to add the prefixation solution to suspended cells dropwise. The fixation solution (2.5% glutaraldehyde in 0.1 M sodium cacodylate buffer, pH 7.4) should also be made immediately before addition to the cell suspension: 1 volume of 25% glutaraldehyde stock solution (Sigma #G5882) is added to 9 volumes of freshly prepared 0.1 M sodium cacodylate buffer (2.14 g of cacodylic acid sodium salt trihydrate (AppliChem #A2140) is dissolved in 100 ml of deionized water, and pH is adjusted to 7.4 with approximately 2 ml of 0.2 M HCl). After every centrifugation step during the fixation procedure, the supernatant should be removed very carefully avoiding disturbing the pellet.

Use the following step-by-step protocol:

• Harvest cells using a cell scraper and suspend them by repeatedly pipetting up and down. Measure the cell density using a cell counter or a hemacytometer, transfer 4–5 × 10^6^ cells into a plastic tube, and spin down at 200 *g* for 5 min in a bucket rotor.
• Pour out the supernatant and completely resuspend the cell pellet in the small amount of medium left (∼100–300 μl) by tapping the tube with a finger.
• Add 1 ml of the prefixation solution dropwise and mix by careful tilting the tube several times.
• Incubate cells in the prefixation solution for 15 min with continuous tilting the tube back and forth (∼100 ° from its vertical position), with a frequency of 20 oscillations per min in an appropriate mixer (e.g., Intelli-Mixer RM-1, ELMI, Latvia).
• Centrifuge cells at 200 *g* for 5 min in a bucket rotor. Remove the supernatant.
• Add 1 ml of the fixation solution and completely resuspend cells by slow and gentle pipetting.
• Incubate cells in the fixation solution for 2 h with continuous tilting the tube back and forth (∼100 ° from its vertical position), with a frequency of 20 oscillations per min.
• Centrifuge cells at 200 *g* for 5 min in a bucket rotor. Remove the supernatant.
• Wash the pellet three times (5 min per wash) without disturbing it with 1 ml of 0.1 M sodium cacodylate buffer (pH 7.4) (see above), and then centrifuge at 200 *g* for 5 min in a bucket rotor.
• Post-fix the pellet for 1 h, continuously shaking (in vertically placed tube, on a regular laboratory horizontal shaker) at 20 rpm in 1% solution of osmium tetroxide in 0.1 M sodium cacodylate buffer (pH 7.4; see above), containing few crystals of potassium ferricyanide (K_3_[Fe(CN)_6_]) that makes the solution yellowish. After this step, no further centrifugation is needed.
• Carefully wash the pellet three times without disturbing it with 1 ml of distilled water and incubate overnight at 4 °C in 1% water solution of uranyl acetate (Serva #77870).

### Drying and embedding in resin

In the second day of the protocol, the cell pellet is washed once with distilled water and then gradually dehydrated it in ethanol series (in 1 ml of 30% and 50% ethanol for 7 min, in 70% and 96% ethanol for 10 min, and in 100% ethanol for 20 min) and then in acetone (twice, in 1 ml for 20 min) continuously shaking at 20 rpm during each incubation, and finally embedded in Agar 100 Resin (Agar Scientific #AGR1031; hereafter resin). The embedding step is crucial for the preparation of the specimen, since the size of the cell pellet is bigger (∼7 mm^3^) than that conventionally used for TEM preparations (∼1 mm^3^). Thus, proper resin infiltration is required.

Use the following step-by-step embedding protocol:

• Incubate the cell pellet in 1 ml of 1:3 (v/v) mixture of resin:acetone for 1 h, with continuous horizontal shaking (in a vertically placed tube on a regular laboratory horizontal shaker) at 20 rpm.
• Remove the solution, add 1 ml of 1:1 (v/v) mixture of resin:acetone, and incubate for 1.5 h with continuous horizontal shaking at 20 rpm.
• Remove the solution, add 1 ml of 3:1 (v/v) mixture of resin:acetone and incubate for 2 h with continuous horizontal shaking at 30 rpm.
• Remove the solution, add 1 ml of pure resin (starting from this step, keep open the lid of the tube) and incubate it for 1–2 h with continuous horizontal shaking at 30 rpm.
• Replace the resin with 1 ml of fresh resin and incubate overnight with continuous horizontal shaking at 30 rpm.
• In the third day of the protocol, replace the resin again with 0.5 ml of fresh resin, and incubate in a desiccator overnight to get rid of bubbles.
• Transfer the specimen to 60 °C for 2–3 days for polymerization of the resin.

### Cutting sections and electron microscopy analysis

Use the following step-by-step protocol:

• Release the polymerized block with the cell pellet from the 1.5-ml tube by cutting its plastic wall with a lancet.
• Fasten the block in a specimen holder and manually cut a few sections with a razor blade from its very tip, so as to reach the cell layer.
• Obtain semi-thin sections (500 nm) using an ultramicrotome (ultracut E, Reihert-Jung, Austria or a similar ultramicrotome) and collect sections on a drop of distilled water placed on a slide; dry the water with a spirit lamp to attach the sections to the slide.
• Stain the sections by covering them with a drop of 1% toluidine blue (Sigma #89640) dissolved in 1% borax (Sigma #71997); heat the drop with a spirit lamp for 1–2 min (without letting them boil or dry completely) and wash them with distilled water.
• Find the region of interest under a light microscope and shape it with a razor blade into a pyramid. Use an ultramicrotome (ultracut UCT, Leica, Austria) to obtain 60 nm sections with a diamond knife (Diatome #DU4515).
• Transfer sections to a 150 mesh hexagonal copper grid (SPI Supplies #2850C-XA) pretreated with acetone for 20–30 min and let them dry for a few hours.
• Sections could be stained with a drop of lead citrate for 4 min to achieve a good contrast for visualization of cellular membranes; however, a good contrast is also obtained using crystals of potassium ferricyanide during post-fixation.
• Cover sections with a thin layer of carbon using a carbon evaporator unit (JEE 4B, JEOL, Japan) to make them conductive and stable under the electron beam.
• Analyze sections under an electron microscope. The images presented here were obtained using a JEM-100SX electron microscope (JEOL, Japan) and a film (Agfa #EB19H).

### Troubleshooting and tips

**Fig. 2:**
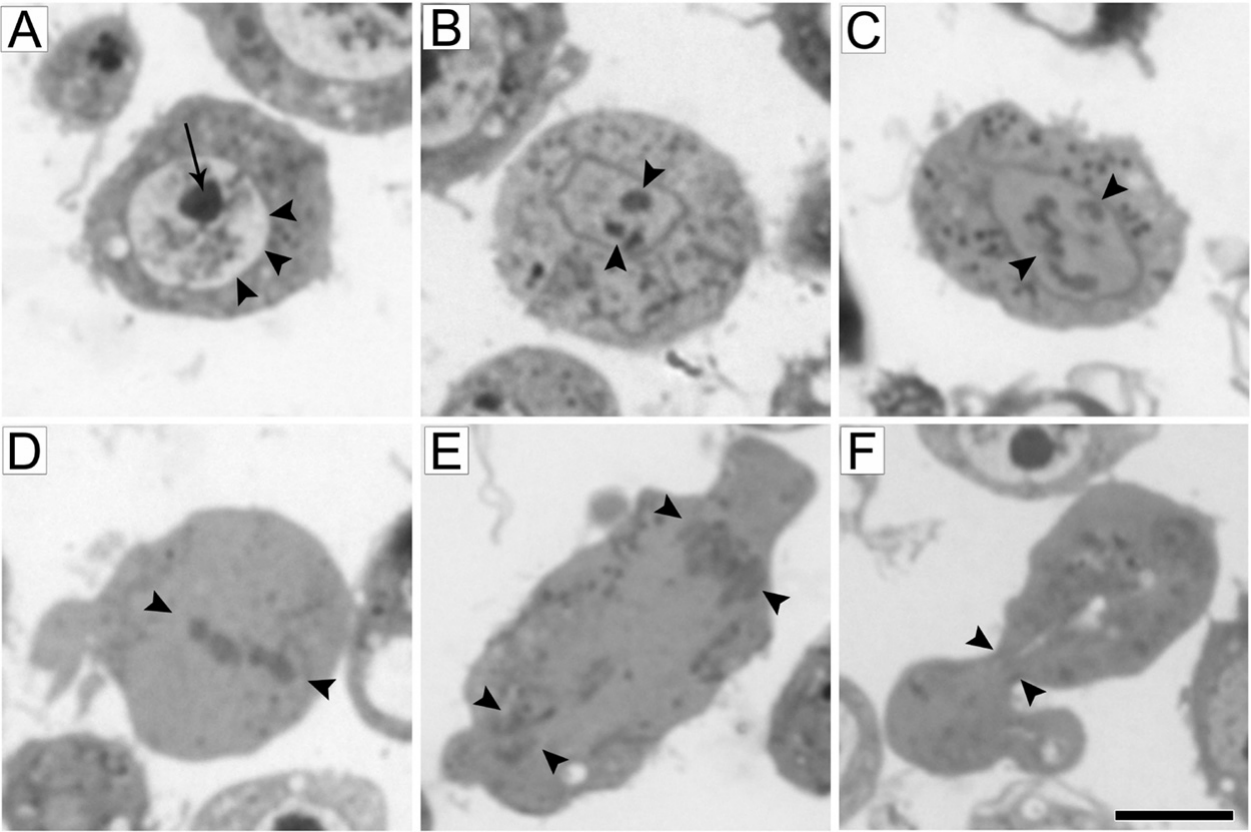
Phases of *Drosophila* S2 cells mitosis observed in semi-thin sections stained with toluidine blue under a light microscope (100× magnification). (A) Interphase, with a light round nucleus (arrowheads) and a dense nucleolus (arrow). (B, C) Late prophase/prometaphase figures showing chromatin condensation (arrowheads) and variations in the nuclear envelope morphology. Note that the nucleus is no longer lighter than the cytoplasm. (D) Metaphase showing well-aligned chromosomes (arrowheads). (E) Anaphase showing two sets of segregating chromosomes (arrowheads). (F) Telophase displaying the cytokinetic constriction and the midbody (arrowheads). Scale bar: 5 μm.

1. Cells clumps in the culture may lead to an irregular cell density in the pellet. Avoid an excessively high density of cells in the culture (not more than 6–8 × 10^6^ cells/ml) to prevent cell clumping (see [7]).
2. The fixation procedure should be gentle but fast; keeping the correct timing of the steps is crucial. Thus, it is advisable to proceed with no more than 4–6 specimens at once.
3. After the first centrifugation, the cell pellet should be carefully resuspended by gentle shaking or pipetting. The prefixation solution should be added dropwise only when the cells are completely resuspended in the small volume of medium left after pouring out the supernatant.
4. Prefixation and fixation solutions must be at room temperature (23 ± 2 °C), as lower temperatures may cause ineffective fixation and depolymerize the microtubules [9], [10].
5. Extensive washing of the cell pellet can remove some cells from the pellet surface. The washing solution should therefore be removed very gently leaving just a little bit of it above the pellet. The new solution should be added very slowly, drop by drop, along the wall of the tube. The pellet should never dry out.
6. The osmium tetroxide penetration into the pellet should be carefully controlled. Sometimes, the bottom of the big (∼7 mm^3^) cell pellet remains unfixed and fails to blacken like the rest of the pellet. In this case, the pellet should be gently flipped over with a toothpick to make the unfixed area accessible to osmium tetroxide. In addition, in this case, the incubation time should be extended up to 15 min.
7. Semi-thin sections can be used for detecting cells at the desired mitotic stage (Fig. 2). Cells in different mitotic phases can be identified by light microscopy from the presence of specific features. We usually stain semi-thin section with toluidine blue. However, mitotic cells might also be detected by phase contrast microscopy in unstained sections. It should be recalled that *Drosophila* mitosis is semi-closed, with the nuclear envelope persisting throughout prometaphase and at least in part also during metaphase [11]. The vast majority of cells are in interphase; they are clearly recognized because of their round and light nucleus with dense nucleoli (Fig. 2A). Cells in late prophase/prometaphase exhibit morphologically irregular (often slightly elongated) nuclear envelopes, an opaque nucleoplasm (similar to the cytoplasm) and condensed chromosomes (Fig. 2B, C). Metaphases are characterized by chromosome alignment at the cell’s equator and a strong reduction, or even the absence, of the nuclear envelope (Fig. 2D). Anaphases are elongated cells containing two groups of segregating chromosomes not surrounded by a nuclear envelope (Fig. 2E). The main feature of telophases is the cytokinetic constriction between the two daughter cells that remain connected by the midbody (Fig. 2F). Thus, light microscopy observation allows the choice of a region of interest in a block, which can then be trimmed to obtain serial sections for TEM analysis. This procedure significantly reduces the time required for collecting the data that are necessary for the ultrastructural analysis of the mitotic phase under study.

## Method validation

**Fig. 3:**
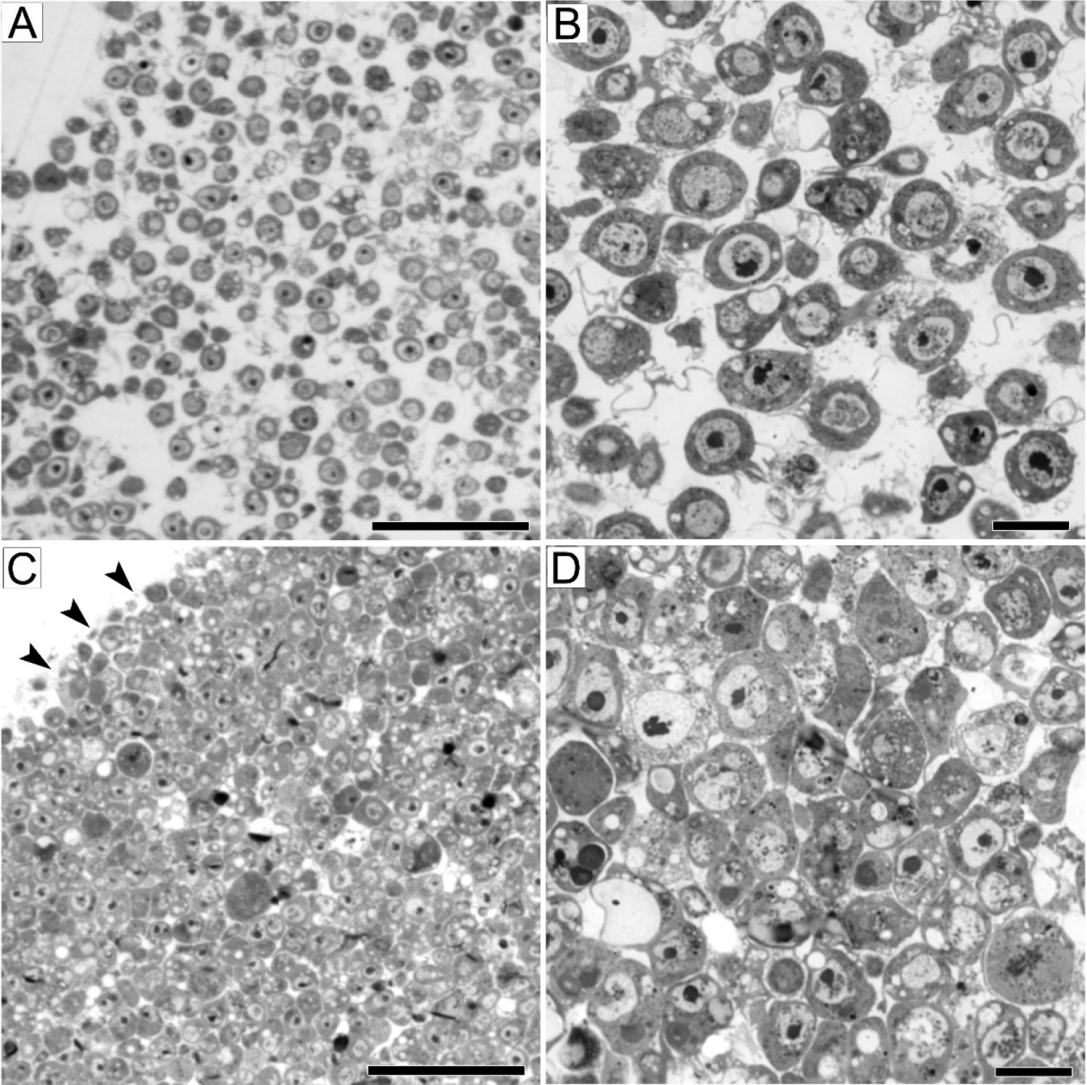
Quality of the cell pellet under a light microscope. Correct (A, B) and incorrect (C, D) embedding of the pellet into the resin. Note the differences in cell densities and the sharp border between the pellet and the resin (arrowheads in C). Scale bars: 50 μm (A, C), 10 μm (B, D).

As mentioned previously, a successful preparation of mitotic cells for TEM analysis depends on the accurate execution of several major steps: fixation, dehydration and embedding in a resin. The quality of embedding is very important and can be checked by the analysis of semi-thin sections of the pellet under a light microscope. In case of proper fixation and infiltration of the resin into the pellet, the cells are well separated and lie at a distance of at least 3–4 μm (Fig. 3A, B). Inaccurate embedding is revealed by a high cell density in the pellet (with distances of less than 1 μm between neighboring cells) and a clearcut border between the cell pellet and the resin (Fig. 3C, D).

**Fig. 4:**
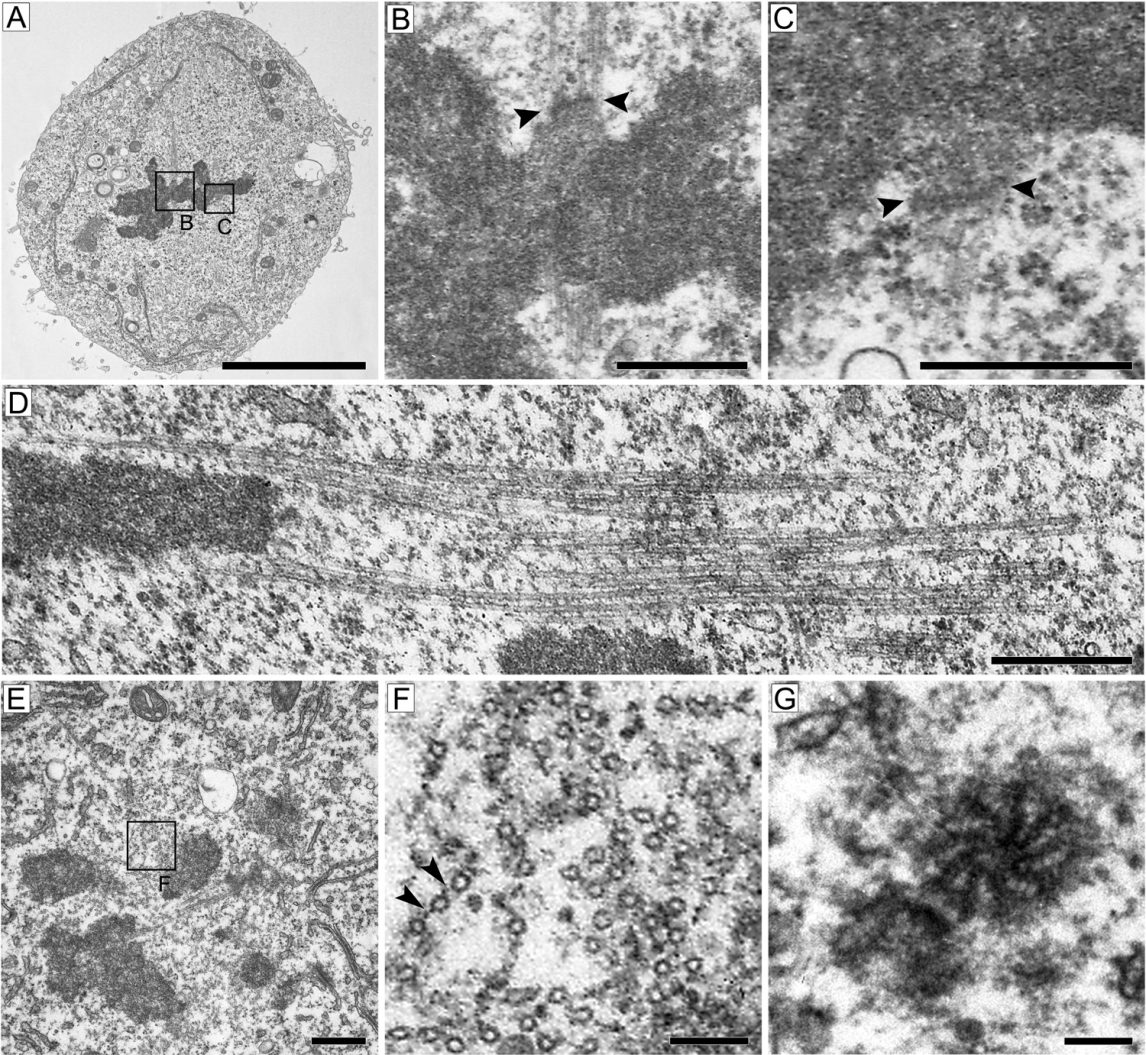
Ultrastructure of *Drosophila* S2 cells undergoing mitosis. (A–C) metaphase; B and C are the enlarged insets indicated in A, showing the ultrastructure of kinetochores (arrowheads) and attached MTs. (D) Fine structure of a kinetochore fiber from a prometaphase. (E) Transverse section of the spindle from a cell in prometaphase. (F) High magnification of MTs from the inset in E; note the proteinaceous bridges (arrowheads) between MTs. (G) Ultrastructure of mother and daughter centrioles. Scale bar: 5 μm (A), 0.5 μm (B–E), 0.1 μm (F, G).

Proper ultrastructure preservation can only be checked under an electron microscope. Dividing cells are easily detected, as they show compact electron-dense chromosome masses and partially disassembled nuclear envelope. As shown in Fig. 4, our protocol provides excellent preservation of fine cellular structures such as microtubules (MTs) and kinetochores. Importantly, sectioning a pellet from a cell suspension provides both longitudinal and transverse sections of the mitotic apparatus, whereas sectioning of an adherent cell monolayer mostly provides longitudinal sections. Transverse sections permit observation and quantification of structural details that are difficult to define in longitudinal sections. For example, transverse sections are particularly useful for visualization of the microtubule bundles that interact with the kinetochores and the proteinaceous structures that connect MTs (Fig. 4E, F).

## Additional information

### Background

*Drosophila* S2 cells serve as an excellent model system for a variety of cells biological analyses and high-throughput screens at genome-wide level [12]. S2 cells are a spontaneously immortalized cell line from a primary culture of *Drosophila* embryonic cells; their characteristics suggest that they are derived from macrophages [6]. S2 cells are very easy to grow and highly susceptible to RNAi. Importantly, in these cells there is no interferon response like in mammalian cells and RNAi can be induced with long double-stranded RNAs (dsRNAs) that are directly incorporated into the cells from the medium. The easy of RNAi-mediated gene silencing has made S2 cells an ideal system for phenotypic analysis of the consequences of inactivation of specific mitotic genes. In addition, S2 cells have been often used to perform genome-wide RNAi-based screens aimed at the identification of new mitotic functions as well as screens aimed at investigating the mitotic functions of selected gene groups, such as those encoding kinesins, actin-binding proteins, kinases or phosphatases (e.g., [2], [13], [14], [15], [16], [17]).

Despite the widespread usage of *Drosophila* S2 cells, only a few studies analyzed their mitotic structures at the ultrastructural level (e.g., [18], [19], [20]). Most protocols for electron microscopy investigation of *Drosophila* cell cultures use flat embedding of cell monolayers [18], [19], [20], [21]. This is an excellent procedure for correlative light-electron microscopy, but is not convenient for analyses that require collection of a large amount of data. Collecting TEM data on multiple mitotic cells is not an easy task, because *Drosophila* tissue culture cells are very difficult to synchronize (e.g., [21], [22]). Here, we describe a straightforward approach for ultrastructural investigation of mitosis in *Drosophila* S2 cells. Our approach is based on pelleting a cell suspension by centrifugation; the cell pellet is then fixed and embedded in resin for TEM analysis. Protocols for correlative light-electron microscopy allow examination of a few dividing cells per specimen [23], whereas sectioning of a cell pellet permits detection of a relatively high number of mitotic cells (∼1 dividing cell per every 100 cells). In addition, since fluorescent imaging can induce cytotoxic and genotoxic effects [24], [25], TEM preparations obtained by correlative light-electron microscopy protocols might reflect some of these effects. Our protocol does not include any treatment of the cells before fixation and usually results in excellent preservation of membranes, microtubules, kinetochores, and cell organelles. In a previous study, it was suggested that centrifugation might cause mechanical stress upon the cells, but this issue was not investigated [21]. We compared the morphologies of intact cells from a monolayer and pelleted cells exposed to mild centrifugation (200*g* for 5 min), and did not find any differences in their ultrastructure (data not shown).

An advantage of our method is that the analysis of semi-thin sections by light microscopy permits selection of cells at the desired mitotic stage, allowing the operator to focus on the process of interest to obtain quantitative information. Another advantage of the method described here is the possibility to observe mitotic spindles from different angles. Cells growing in a monolayer usually form spindles with the long axis parallel to the substrate and therefore most sections of these spindles are longitudinal. In contrast, sectioning the pellets provides many transverse sections of the spindle. An analysis of transverse section is particularly useful to define the arrangement of MTs and their interaction with kinetochores.
